# Apolipoprotein E4 and Insulin Resistance Interact to Impair Cognition and Alter the Epigenome and Metabolome

**DOI:** 10.1038/srep43701

**Published:** 2017-03-08

**Authors:** Lance A. Johnson, Eileen Ruth S. Torres, Soren Impey, Jan F. Stevens, Jacob Raber

**Affiliations:** 1 Department of Behavioral Neuroscience, Oregon Health & Science University, Portland, OR 97239, USA; 2 Oregon Stem Cell Center and Department of Pediatrics, Oregon Health & Science University, Portland, OR 97239, USA; 3 Department of Cell, Developmental and Cancer Biology, Oregon Health & Science University, Portland, OR 97239 USA; 4 Department of Pharmaceutical Sciences, Oregon State University, Corvallis, OR 97331 USA; 5 Linus Pauling Institute, Oregon State University, Corvallis, OR 97331 USA; 6 Departments of Neurology and Radiation Medicine, Division of Neuroscience, ONPRC, Oregon Health & Science University, Portland, OR 97239 USA

## Abstract

Apolipoprotein E4 (E4) and type 2 diabetes are major risk factors for cognitive decline and late onset Alzheimer’s disease (AD). E4-associated phenotypes and insulin resistance (IR) share several features and appear to interact in driving cognitive dysfunction. However, shared mechanisms that could explain their overlapping pathophysiology have yet to be found. We hypothesized that, compared to E3 mice, E4 mice would be more susceptible to the harmful cognitive effects of high fat diet (HFD)-induced IR due to apoE isoform-specific differences in brain metabolism. While both E3 and E4 mice fed HFD displayed impairments in peripheral metabolism and cognition, deficits in hippocampal-dependent spatial learning and memory were exaggerated in E4 mice. Combining genome-wide measures of DNA hydroxymethylation with comprehensive untargeted metabolomics, we identified novel alterations in purine metabolism, glutamate metabolism, and the pentose phosphate pathway. Finally, in E4 mice, the metabolic and cognitive deficiencies caused by HFD were rescued by switching to a low fat diet for one month, suggesting a functional role was associated with reversal of the same metabolic pathways described above. These results suggest a susceptibility of E4 carriers to metabolic impairments brought on by IR, and may guide development of novel therapies for cognitive decline and dementia.

Apolipoprotein E4 (E4) and type 2 diabetes (T2D) share pathophysiological features that are linked to cognitive impairment and dementia. However, it is unclear if these two critical risk factors interact to worsen cognitive decline, and the shared mechanisms that could explain their overlapping pathology have yet to be found.

The most significant genetic risk factor for late onset Alzheimer’s disease (AD) is E4^1^. The *APOE* gene encodes three major isoforms in the human population (E2, E3, and E4)^2^. In the periphery, apoE is associated with circulating lipoproteins, primarily very low density lipoproteins (VLDL) and high density lipoproteins (HDL), while the brain synthesizes its own pool of apoE, the majority of which are secreted by astrocytes^3^. In addition to well-established effects on AD pathology, apoE also shows substantial isoform-specific effects on metabolism^4^.

Obesity and diabetes have reached epidemic proportions worldwide^5^. Aside from traditional complications, obesity, insulin resistance (IR) and T2D pose additional health risks in the form of cognitive dysfunction and dementia. Compared with non-diabetic individuals, those with diabetes have a ∼70% greater risk for the development of vascular dementia or AD^6^. Obesity and IR are also associated with decreased cognitive function and an increased risk of dementia, even in the absence of overt diabetes^7^. Finally, AD itself is associated with increased incidence of diabetes^8^, and the brains of patients with mild cognitive impairment (MCI) and AD are functionally insulin resistant^9^.

E4 and T2D also appear to act synergistically to drive cognitive dysfunction and increase the risk of AD and vascular dementia^10^,^11^. E4 and T2D share characteristics with established links to neurodegeneration. For instance, while glucose hypometabolism is a core feature of AD, E4 itself has also been associated with lower rates of cerebral glucose metabolism^12^. Several other pathways that are negatively influenced by brain insulin levels are similarly altered in E4 + individuals, including amyloid β clearance, neuroinflammation, and synaptic dysfunction^3^,^13^.

A primary, modifiable contributor to obesity, IR and T2D is caloric excess, particularly in the form of high intake of saturated fats and high glycemic index foods. Consumption of this Western style diet increases the risk of dementia, and the effects may be modulated by *APOE* genotype^14^. However, examining the interaction between E4 and diet in humans is difficult due to considerable individual variations in dietary habits and differences in genetic factors other than *APOE*. Therefore, we employed a mouse model of human apoE to study the interaction of E4 with high fat diet (HFD)-induced IR on brain metabolism and cognitive function, and to identify potential pathways driving this interaction. Through the integration of genome-wide measures of epigenetic changes and untargeted metabolomics, we highlight several novel and interconnected metabolic pathways that may underlie the observed cognitive impairments.

## Results

### HFD results in glucose intolerance in E3 and E4 mice; reduced weight gain in E4 mice

**Figure 1:**
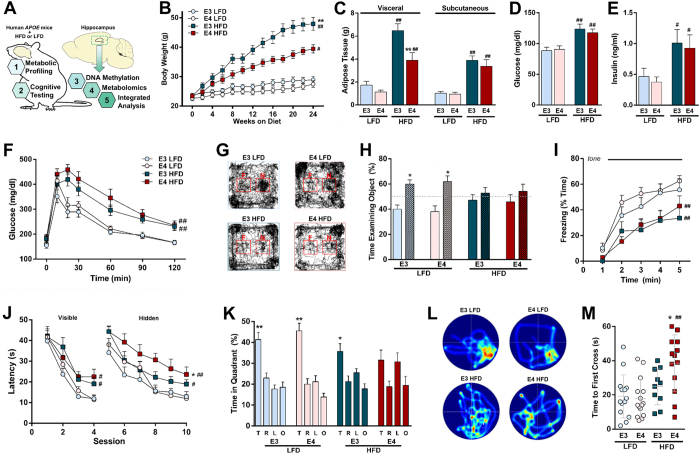
HFD-induced metabolic and cognitive impairments in spatial memory more pronounced in E4 than E3 mice. (**A**) Peripheral metabolism and cognitive function were assessed following six months of HFD or LFD. Genome wide DNA hydroxymethylation, untargeted metabolomics, and an integrated analysis of the two were performed on isolated hippocampal tissue. (**B**) E4 HFD mice gain less weight. (*n* = 12–19) (**C**) E4 HFD mice accumulate less visceral adipose tissue following 6 months of HFD. (*n* = 12–14). (**D-E**) Chronic HFD induces hyperglycemia and hyperinsulinemia. Blood glucose (**D**) and plasma insulin (**E**) were measured following an overnight fast. (C, *n* = 17–24; D, *n* = 8–14). (**F**) Glucose intolerance is exaggerated in E4 HFD mice. Mice were administered an oral gavage of glucose following a four hour fast, and area under the curve was calculated. (*n* = 10–14). (**G-H**) Object recognition, 24 hours after training, is impaired in E3 HFD and E4 HFD mice. Familiar = open bars, novel = checkered bars. Representative tracks are shown in G. (*n* = 10–14). (**I**) Cued fear memory, 24 hours after training, is impaired in HFD mice. (*n* = 10–14). (**J**) E4 worsens HFD-induced deficits in spatial learning and memory in the water maze. Latency to locate a visible (left) or hidden (right) escape platform during the water maze. (*n* = 10–14). (**K-M**) HFD-induced deficits in long-term spatial memory are more profound in E4 mice. Long-term spatial memory was measured as the percent time spent searching in the target quadrant in a 72 hour probe trial (**K**). Representative heat maps (platform location right bottom quadrant) show deficits in search strategies in E4 HFD mice (**L**). The average time at which the mice first cross the target location is longer in E4 HFD mice (**M**). (*n* = 10–14) *p < 0.05, **p < 0.01 compared to E3; #p < 0.05, ##p < 0.01, compared to LFD (*B, F, I, J,* repeated measures ANOVA) (*C-E, H, M,* ANOVA followed by Tukey’s multiple comparison test). For *K*, *p < 0.05, **p < 0.01 Target quadrant compared to all other quadrants (ANOVA followed by Tukey’s multiple comparison test). T, target; R, right; L, left; O, opposite. Error bars represent mean ± SEM.

After six months of HFD or LFD, we measured markers of peripheral metabolism, evaluated cognitive function, and analyzed the hippocampal epigenome and metabolome in human apoE mice (Fig. 1A). HFD led to increases in body weight and adipose tissue mass in both groups. However, E4 mice gained less weight and less visceral adipose tissue than E3 mice (Fig. 1B–C). Higher measures of glucose intolerance have previously been negatively associated with cognitive function in humans^15^, and this relationship may be modified by apoE isoform^14^,^16^,^17^. However, fasting glucose and insulin levels were similarly elevated in E3 HFD and E4 HFD mice (Fig. 1D–E), and both E3 HFD and E4 HFD mice showed delayed glucose clearance following an oral challenge compared to LFD controls (Fig. 1F).

### HFD-induced impairments in spatial learning and memory, but not in object recognition or cued fear memory, are exaggerated in E4 mice

To assess cognitive function, we first examined object recognition (Fig. 1G–H). When introduced to a ‘familiar’ and a ‘novel’ object, E3 LFD and E4 LFD mice showed a robust preference for the novel object but neither E3 HFD nor E4 HFD mice did, indicating impaired recognition memory (Fig. 1G–H). We next assessed cued fear learning and memory. All groups showed similar acquisition of fear, shock response, and baseline motion during training (Figure S1A–C). However, both E3 HFD and E4 HFD mice showed decreased freezing when reintroduced to the tone 24 hours later (Fig. 1I), indicating impaired cued fear memory.

We next assessed spatial learning and memory in the water maze. Despite similar swim speeds (Figure S1D), HFD mice required more time to reach a clearly marked platform than LFD mice (Fig. 1J, visible), indicating difficulties in general task learning. When the platform was hidden from view, E4 HFD mice showed significantly worse learning curves compared to E4 LFD and E3 HFD mice (Fig. 1J, hidden). To test long-term memory retention, we conducted probe trials (no platform) at 24 (Figure S1E–F) and 72 (Fig. 1K) hours post-training. E3 HFD and E4 HFD mice showed impaired spatial memory retention in both probe trials. While E3 HFD mice showed spatial memory retention following additional training, E4 HFD mice did not (Fig. 1K). Representative heat maps tracking search strategies, as well as the time to first cross the platform location, also showed clear deficits in E4 HFD mice (Fig. 1L–M). Thus, HFD-induced deficits in hippocampus-dependent spatial learning and memory are more pronounced in mice expressing E4 than E3.

These cognitive deficits appeared specific to HFD-induced IR, as despite severe hyperglycemia (>400 mg/dl fasting glucose), there were no significant cognitive impairments in either E3 or E4 mice in a separate cohort of mice with type 1 diabetes (T1D) (Figure S2).

### E4 HFD mice show a unique profile of DNA hydroxymethylation in the hippocampus

**Figure 2:**
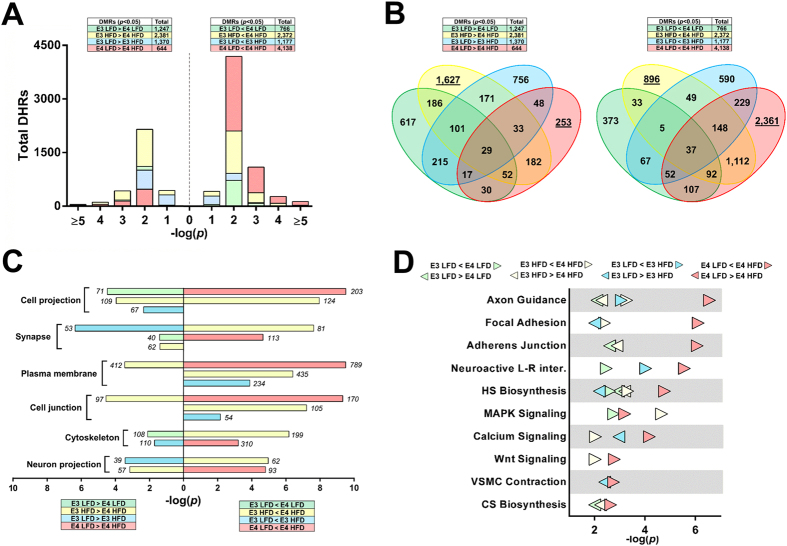
E4 HFD mice show a unique profile of DNA hydroxymethylation in the hippocampus. (**A**) A large number of DHRs are hypermethylated in E4 HFD compared to E4 LFD mice. Histogram of differentially hypo-and hypermethylated regions for each comparison (FDR-adjusted -log[*p* < 0.05]). (For *A-D*, n = 10 pools; 2 pools/experimental group, consisting of 4–6 individual DNA samples/pool). (**B**) Many DHRs are uniquely hypermethylated in E4 HFD mice. Venn diagrams depict directional overlap between differentially hydroxymethylated regions. Boxes show total number of DHRs for each comparison (FDR-adjusted -log[*p* < 0.05). Underlined numbers represent DHRs that were uniquely altered in E4 HFD mice. (**C**) Gene ontology shows enrichment in cell component categories specific to neurons. Bar graph depicts significantly enriched gene ontology terms in the indicated comparisons of DHRs based on a cellular component subset (number of significantly altered DHRs in each category are shown in *italics*). (**D**) Multiple pathways related to neuronal function, vascular physiology, cell adhesion, proteoglycan synthesis, and signaling are hypermethylated in E4 HFD mice. Categories represent the top 10 most significantly enriched gene ontology terms for the indicated comparisons of DHRs based on KEGG pathways. CS, chondroitin sulfate; HS, heparin sulfate; Neuroactive L-R inter. neuroactive ligand-receptor interaction; VSMC, vascular smooth muscle cell.

Alterations in DNA methylation may contribute to the pathogenesis of T2D and its complications^18^. 5′-hydroxymethylated DNA (5hmC) appears especially important for cognitive processes, and changes in 5hmC strongly correlate with transcriptionally active regions^19^,^20^. Because the E4-exaggerated cognitive deficits were hippocampus-dependent, we performed an unbiased, genome-wide analysis of 5hmC in the hippocampus of E3 and E4 mice. The global distribution of epigenetic marks was similar among groups, as accumulation of 5hmC in intragenic, intergenic, exonic, and untranslated regions did not significantly differ (Figure S3A). Although global distributions of 5hmC signal were not altered, thousands of differentially hydroxymethylated regions (DHR) were significantly affected by apoE isoform and/or diet. The most dramatic changes in 5hmC levels were observed between comparisons of E4 HFD vs E4 LFD (4,782 total DHRs) or E3 HFD mice (4,753 total DHRs) (Fig. 2A–B). Furthermore, a large number of the DHRs were unique to E4 HFD mice. For example, more than half of the DHRs in E4 HFD mice were uniquely hypomethylated compared to E3 HFD mice (1,627/2,381), or hypermethylated compared to E4 LFD mice (2,361/4,138) (Fig. 2B, bold).

To identify putative biological functions for the DHRs, we performed gene ontology (GO) analyses. Our analyses revealed numerous cellular component GO categories specific to neurons, including synapse and neuron projection (Fig. 2C). The most substantial changes, both in significance and number of pathways affected, were seen in E4 HFD mice. They included terms related to neuron differentiation, axonogenesis, intracellular signaling, nucleic acid regulation, and phosphate metabolism (Figure S3B). We next compared DHRs using a KEGG pathway-based GO analysis. Again, the most significant changes occurred in E4 HFD mice, and involved pathways related to neuronal function, vascular physiology, cell adhesion, proteoglycan synthesis, and signaling (Fig. 2D). Additionally, several genes known to co-regulate multiple pathways listed above were uniquely hydroxymethylated in E4 HFD mice (Figure S3C). Thus, compared to E3, E4 mice show an exaggerated response and a unique pattern of neuronal-associated changes in hippocampal 5hmC when challenged with a HFD.

GO analyses for DHRs within 10 kb of a transcriptional start site did not identify significant categories. Analyses using a 20 kb window identified the following significant categories (p < 0.001): E3 LFD vs E3 HFD downregulated – membrane, protein binding, and cytoplasm; E4 LFD vs E4 HFD downregulated – membrane, positive regulation of transcription, ion transport; E3 HFD vs E4 HFD downregulated – protein binding, nucleus, cytoplasm. This suggests that proximal DHRs are associated with general cellular and/or metabolic processes.

### ApoE isoform-dependent changes in the hippocampal metabolome

**Figure 3:**
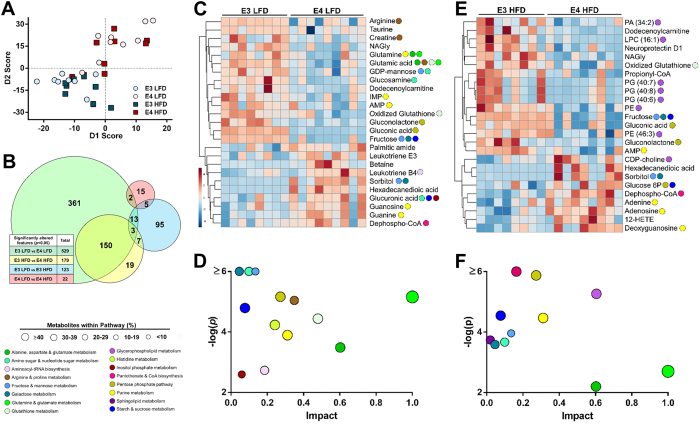
*APOE* genotype substantially alters the hippocampal metabolome. (**A**) Principal component analysis (PCA) score plot of the hippocampus metabolome shows distinct separation based on apoE isoform. (*n* = 8–9). (**B**) The majority of altered features differ by apoE isoform. The Venn diagram depicts overlap between significantly altered MS/MS features (*p* < 0.05 FDR adjusted). The box shows the total number of altered features for each comparison. (*n* = 8–9). (**C**–**F**) Multiple metabolic pathways are significantly altered in an apoE isoform-dependent fashion. Hierarchical clustering of the top 25 most significantly altered metabolites between E3 LFD and E4 LFD mice (**C**), or between E3 HFD and E4 HFD mice (**E**). Color in the heat map reflects the relative metabolite abundance level, with red being higher, and blue lower, than the mean value. Colored circles denote the metabolic pathway(s) in which each metabolite plays a role. A global view of the metabolome was created using a pathway impact analysis (**D,F**), which reflects key nodes in pathways that have been significantly altered in an apoE isoform-dependent fashion in LFD (**D**) or HFD (**F**) fed mice. The y-axis shows significance based on pathway enrichment, the x-axis shows impact based on a topology measure of centrality and connectedness, and circle size reflects the percentage of all metabolites within a given pathway that are represented. (*n* = 8–9). Abbreviations: 12-HETE, 12-Hydroxyeicosatetraenoic acid; AMP, Adenosine monophosphate; CoA, Coenzyme A; FADH, Flavin adenine dinucleotide (semiquinone); G6P, Glucose 6-phosphate; GDP, Guanosine diphosphate; GSSG, Glutathione disulfide; IMP, Inosine monophosphate; LPC, Lysophosphatidylcholine; NAGly, N-Arachidonoyl glycine; PA, Phosphatidic acid; PE, Phosphatidylethanolamine; PG, Phosphatidylglycerol.

Employing untargeted metabolomics, we next detected and relatively quantified 12,464 unique MS/MS features within the hippocampus of E3 and E4 mice. When evaluated using a supervised principal components analysis, the overall profile of E4 mice was shifted from that of E3 mice, regardless of diet (Fig. 3A). The majority of significantly altered features were due to apoE isoform (Fig. 3B). Using mass, retention time and MS/MS spectra, we confirmed the identity of 138 unique metabolites (Table S1), of which 58 were significantly altered by apoE isoform and/or diet (Figure S4A). Heat maps showing hierarchical clustering of altered metabolites between E3 and E4 mice revealed distinct patterns of change (Fig. 3C,E). Among these metabolites, components of purine, various sugar (sucrose, galactose, fructose and mannose), and glycerophospholipid (GPL) pathways were most highly represented. While some overlap existed, a HFD altered distinct pathways in E3 and E4 mice: GPL and sphingolipid metabolism in mice with E3, and coenzyme A (CoA) biosynthesis and the pentose phosphate pathway (PPP) in mice with E4 (Figure S4B–E). Numerous pathways were altered in an apoE isoform-dependent fashion. In LFD mice, the pathways most significantly altered by apoE isoform were fructose, mannose and galactose metabolism, amino sugar and nucleotide sugar metabolism, glutamate metabolism, and the PPP (Fig. 3D). In HFD mice, the most significantly altered pathways included pantothenate and CoA biosynthesis, the PPP, GPL metabolism, starch and sucrose metabolism, and purine metabolism (Fig. 3F). In both LFD and HFD mice, the pathway most impacted by apoE isoform based on a measure of centrality was glutamate metabolism.

### Integration of DNA hydroxymethylation and metabolomics analyses reveals alterations in three novel and interconnected metabolic pathways

**Figure 4:**
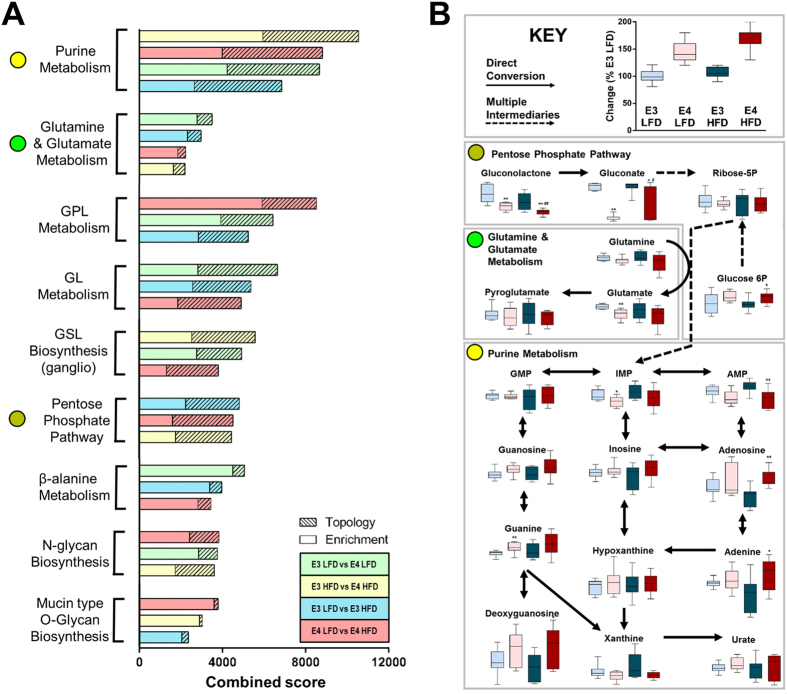
Integration of unbiased epigenetic and metabolomics analyses reveals alterations in several novel and interconnected metabolic pathways. (**A**) An integrated pathway analysis shows that multiple pathways are significantly altered by both apoE isoform and HFD. The analysis integrates changes in metabolite concentrations with alterations in DNA hydroxymethylation of genes associated with DHRs. The enrichment score evaluates whether the metabolites and genes in a particular pathway are overrepresented compared to random chance based on a hypergeometric analysis, while the topology score estimates the biological impact of a given gene or metabolite based on its position within a pathway. (For DNA hydroxymethylation, n = 10 pools; 2 pools/experimental group, consisting of 4–6 individual DNA samples/pool; for metabolomics, n = 8–9). (**B**) Multiple metabolites related to purine metabolism, glutamate metabolism, and the pentose phosphate pathway are significantly altered by apoE isoform and/or HFD diet. Arrows note connected metabolites within each simplified metabolic pathway, while box and whisker plots show metabolite concentrations relative to E3 LFD.

DNA methylation and metabolism are tightly linked, with crosstalk at the molecular level^21^. To determine the overlap in pathways identified by the 5hmC and metabolomics analyses, we integrated the data using a platform that maps changes against established KEGG pathways. The resulting integrative analysis revealed multiple pathways that were significantly altered by both apoE isoform and HFD. They included changes related to glycerolipid metabolism and glycan biology (Fig. 4A). Of particular interest due to their biological relevance and interconnectivity are the PPP and the two pathways with the highest combined enrichment and topology scores: purine metabolism and glutamate metabolism (Fig. 4A–B). Within these three pathways, we identified multiple individual metabolites which were altered by apoE isoform and/or HFD. They included adenosine monophosphate (AMP), adenosine, and adenine within the purine metabolism pathway, gluconolactone, gluconate, and glucose 6-phosphate within the PPP, and glutamate (Fig. 4B).

### A dietary intervention rescues HFD-induced impairments in E4 mice and reverses alterations in the hippocampal epigenome and metabolome

**Figure 5:**
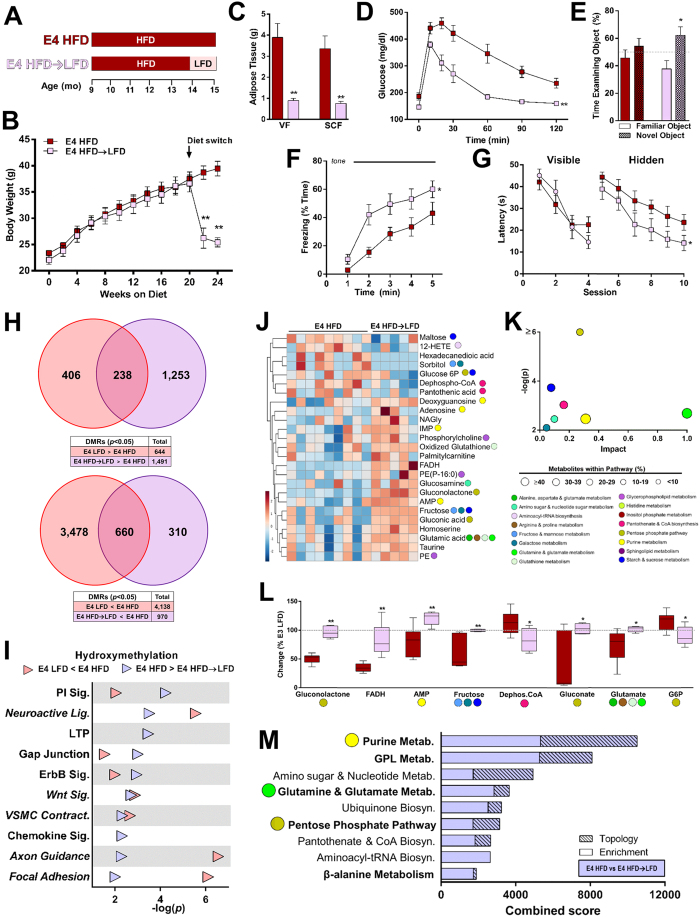
A dietary intervention rescues HFD-induced metabolic and cognitive impairments in E4 mice and reverses epigenetic and metabolic changes. (**A**) A subset of E4 mice were fed HFD for 5 months, followed by LFD for one month (E4 HFD → LFD). (**B-C**) E4 HFD → LFD mice lose body weight and adipose tissue following the switch to a LFD. Body mass (**B**), and visceral fat (VF) and subcutaneous fat (SCF) pads (**C**) were weighed. (*n* = 9–19). (**D**) Glucose tolerance improves following the dietary intervention. Mice were administered an oral gavage of glucose following a 4 hour fast. (*n* = 9–13). (**E**) Object recognition is rescued in E4 HFD → LFD mice. (*n* = 7–12). (**F**) Cued fear memory is rescued in E4 HFD → LFD mice. (*n* = 7–12). (**G**) Spatial learning and memory in the water maze is rescued in E4 HFD → LFD mice. (*n* = 7–12). (**H**) Multiple DHRs are similarly altered in E4 LFD and E4 HFD → LFD, compared to E4 HFD mice. The boxes show total number of DHRs for each comparison (*p* < 0.05). (n = 4 pools; 2 pools/experimental group, consisting of 5 individual DNA samples/pool). (**I**) The majority of pathways differentially hydroxymethylated in E4 LFD mice are similarly altered in E4 HFD → LFD mice. (n = 4 pools; 2 pools/experimental group, consisting of 5 individual DNA samples/pool). (**J-K**) The E4 HFD metabolome is significantly altered by a dietary intervention. Hierarchical clustering of the top 25 most significantly altered metabolites between E4 HFD and E4 HFD → LFD mice (**J**). Pathway impact analysis (**K**). (n = 5–9). (**L**) Multiple metabolites are significantly altered following the dietary intervention (p < 0.05, t-test). Box and whisker plots reflect metabolite concentrations relative to E3 LFD. (n = 5–9). (**M**) An integrated pathway analysis shows that the dietary intervention affects, among others, the three pathways highlighted in Figure 5 (purine metabolism, glutamate metabolism, and PPP). *p < 0.05, **p < 0.01 compared to E4 HFD (*C,E,* ANOVA followed by Tukey’s multiple comparison test; *B,D,F,G,* repeated measures ANOVA). For *E*, *p < 0.05, compared to Familiar Object (ANOVA followed by Tukey’s multiple comparison test). T, target; R, right; L, left; O, opposite. Error bars represent mean ± SEM. E4 HFD data is reproduced from Figures 1, 2, 3, 4, 5.

As E4 mice were particularly sensitive to the detrimental metabolic and cognitive effects of a HFD, we next asked whether these effects were reversible. An independent group of E4 HFD mice were administered a LFD for one month following five months of HFD (hereafter referred to as E4 HFD → LFD) (Fig. 5A). Immediately following the switch to a LFD, E4 HFD → LFD mice lost substantial body weight and fat mass (Fig. 5B–C). The switch was associated with an improvement in glucose tolerance (Fig. 5D), and a complete rescue of cognitive function. E4 HFD → LFD mice showed robust object recognition (Fig. 5E), and despite similar learning curves and rates of generalized fear (Figure S5A–C), they showed enhanced freezing in response to a tone, suggesting a rescue of cued fear memory (Fig. 5F). E4 HFD → LFD mice also showed significant improvements in hippocampus-dependent spatial learning and memory (Fig. 5G), including long-term memory retention in the water maze (Figure S5E–H).

We next determined whether the observed rescue of cognitive function was associated with a reversal of HFD-induced changes in the hippocampal epigenome and metabolome. Multiple DHRs that were hypermethylated in E4 HFD vs E4 LFD mice were similarly altered in the E4 HFD vs E4 HFD → LFD comparison (Fig. 5H), indicating that many of the 5hmC changes associated with a HFD were reversed following the dietary intervention. The Venn diagrams depict directional overlap between differentially hydroxymethylated regions. There was also substantial biological overlap in 5hmC patterns at the individual gene level (Table S2), as well as a majority of KEGG pathways differentially hydroxymethylated in E4 LFD mice being similarly altered in E4 HFD → LFD mice (Fig. 5I). Categories represent the top 10 most significantly enriched KEGG pathways for the comparison of DHRs between E4 HFD and E4 HFD → LFD mice (FDR-adjusted *p* < 0.05). Likewise, the most impacted metabolic pathways included the PPP, starch and sucrose metabolism, CoA biosynthesis, purine metabolism, and glutamate metabolism (Fig. 5J–K). Furthermore, multiple individual metabolites – the vast majority belonging to the aforementioned pathways – were significantly altered by the HFD → LFD intervention, with all returning to similar levels as E3 LFD controls (Fig. 5L). Finally, an integrated pathway analysis of the 5hmC and metabolomics data showed that the HFD → LFD intervention affected, among others, the three previously highlighted pathways: purine metabolism, glutamate metabolism, and the PPP (Fig. 5M). The analysis integrates changes in metabolite concentrations with alterations in hydroxymethylation of genes associated with DHRs. Pathways in bold are significantly altered in both the E4 HFD vs E4 LFD and E4 HFD vs E4 HFD → LFD comparisons. These results point to a functional role for the aforementioned pathways, as they are significantly altered in an apoE isoform-dependent fashion by both HFD and the HFD → LFD dietary intervention.

## Discussion

The pathologies of diabetes and dementia are strongly linked and both share features with the harmful processes associated with E4, the strongest genetic risk factor for late onset AD. However, the shared mechanisms that underlie this important connection have yet to be found. In the current study, we examined the effects of E4 on metabolism and cognitive function in a mouse model of HFD-induced IR using an unbiased and integrative approach for profiling metabolism within the hippocampus.

Interestingly, the cognitive impairments we observed in HFD fed E3 and E4 mice were not observed in a model of Type 1 Diabetes (T1D) – as we have previously observed in wildtype mice^22^ – suggesting that these negative cognitive effects are specific to the background of HFD-induced obesity and IR. However, many studies have demonstrated cognitive dysfunction in rodent models of T1D,^23^ and discrepancies with our results could be due to a number of factors including mouse age, sex and strain, the specific cognitive tasks employed, and delivery method of STZ (intracerebroventricular vs intraperitoneal vs intravenous). Still, our data are consistent with human studies where learning and memory are generally spared in T1D patients, while multiple memory deficits are noted even in the early stages of T2D^24^,^25^.

On the other hand, E4 and T2D share several neuropathological features^3^,^13^ including alterations in brain metabolism. Metabolic deficits are present decades in advance of AD onset. For instance, in the case of glucose hypometabolism, reductions in cerebral glucose utilization are observed in normal E4 + volunteers as young as their 20 s^12^, and regional patterns of hypometabolism are not associated with amyloid deposition^26^. Expression of molecules involved in insulin signaling are altered in E4 mouse and human brains^27^,^28^. Given the overlap, several anti-diabetic agents have been tested for treatment of AD, with many showing reduced benefits in E4 + individuals^29^. However, despite the evidence linking T2D and E4, very few studies have explored the potential interaction of these two critical risk factors^30^,^31^.

Altered patterns of DNA methylation are present in tissues of individuals with T2D^18^ and AD^32^. In particular, regulation of 5hmC might modulate hippocampus-dependent cognitive processes. High levels of 5hmC are found in the CNS relative to other tissues, particularly in the hippocampus^33^. In both humans and mice, hippocampal function appears to be especially sensitive to the effects of a Western diet^34^. The exaggerated cognitive impairment in E4 HFD mice was hippocampus-dependent, and associated with epigenetic alterations.

**Figure 6:**
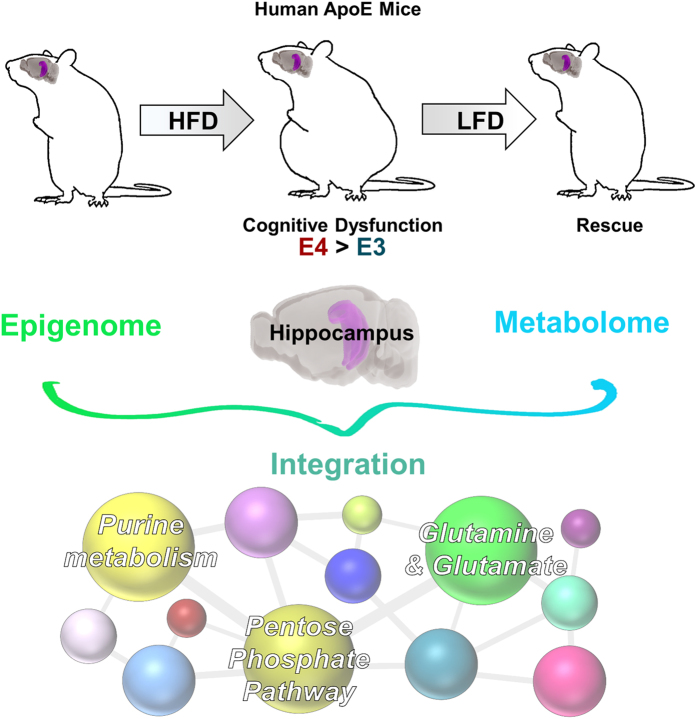
Integrated epigenome-metabolome analysis revealed roles for purine metabolism, the pentose phosphate pathway, and glutamine and glutamate. For details, see text.

DNA methylation and metabolic pathways are highly responsive to environmental cues and each other. For instance, the caloric and micro-nutrient composition of the diet influences methylation status, while various metabolic pathways are controlled at the epigenetic level^21^. However, an analysis at each level independently is likely insufficient to fully characterize a complex biological system. Thus, we integrated the two ‘omics platforms to reveal several intriguing pathways which were altered by E4 and HFD. Of particular interest due to their relative novelty and biological relevance were three interconnected metabolic pathways: purine metabolism, glutamate metabolism, and the PPP (Fig. 6).

Although the purine metabolic pathway has been associated with the development of diabetic microvascular complications, very little is known about its involvement in AD. Purine metabolism is deregulated during AD in a manner that is dependent both on brain region and stage of disease^35^,^36^,^37^. Observed changes in adenosine in E4 HFD mice could be secondary to changes in AMP usage, or may alternatively reflect a vascular response. The latter scenario is intriguing given that several purines have vasoreactive properties, and that purine metabolites accumulate (and glucose oxidation via the PPP increases) during cerebral hypoxia in mammals^38^,^39^. Further, our 5hmC analyses also pointed to vascular and/or BBB dysfunction as areas potentially affected by E4 and HFD, with enrichment in pathways related to focal adhesion, adherens junction, and vascular smooth muscle cell contraction. E4 has been associated with BBB breakdown in multiple studies^40^, and because T2D and E4 are key risk factors for both AD and vascular disease, BBB dysfunction and cerebral hypoxia may represent a shared pathology.

The second major metabolic pathway centers on glutamate, the most abundant excitatory neurotransmitter in the CNS. Glutamate plays a vital role in brain energy metabolism, linking glucose utilization to neuronal activity, and is produced during the first committed, regulated step in the pathway of *de novo* purine biosynthesis, thereby also coupling the PPP with purine metabolism. Similar to our findings, Dumanis *et al*. showed that E4 mice have decreased levels of glutamate compared to mice with E3^41^. E4 has also been shown to impair glutamate receptor function by affecting intracellular trafficking^42^, and increases in glutamate receptors have been described in E4 mice^41^.

The integrated analysis also highlighted a novel potential role for the PPP. Aside from supplying ribose-5-phosphate for purine nucleotide biosynthesis, the PPP plays a crucial role in maintaining cellular redox through regeneration of NADPH. Although the PPP remains largely unexplored in the background of neurodegenerative disease, two studies have linked PPP enzymatic activity and oxidative stress to AD^43^,^44^. Interestingly, the role of the PPP in cerebral glucose metabolism and redox maintenance draws some intriguing parallels to cancer biology. Unlike normal tissues, cancer cells rely heavily on aerobic glycolysis, a phenomenon known as the Warburg effect^45^. There is a new appreciation for the substantial link between aerobic glycolysis, redox maintenance and the regulation of blood flow in the brain. In fact, recent work even suggests that, like cancer cells, neurons display an adaptive advantage for survival by managing their redox state via the PPP^46^. Further, the areas of the normal brain that demonstrate the highest rates of aerobic glycolysis show near complete overlap with areas of the AD brain that preferentially accumulate amyloid, and it has thus been suggested that impairments in aerobic glycolysis may contribute to AD pathophysiology^47^.

Importantly, several of the metabolic pathways we identified as being altered by E4 and HFD have also been identified in the brain tissue of AD patients compared to controls, including amino sugar and nucleotide sugar metabolism, galactose metabolism, fructose and mannose metabolism, and the PPP^48^. Notably, these pathways represent secondary (non-glucose) sources of energy for neurons. In addition to the cerebral glucose hypometabolism in E4 + individuals, a global metabolic shift toward increased lipid oxidation – at the expense of glucose metabolism – was recently described in E4 mice^49^. Thus, perhaps an inherent inability of E4 + individuals to use glucose is reflected in the sum of these findings; although whether it occurs at the level of the BBB or cellular uptake, oxidative phosphorylation, aerobic glycolysis or elsewhere remains unclear. Adding further predictive value, the only metabolic pathway significantly altered in MCI patients who progressed to AD vs those who did not, was the PPP^44^. Thus, the PPP may represent a novel target which potentially ties E4 to pathological changes in glucose metabolism, oxidative stress and neuronal survival.

Finally, because E4 HFD mice were most affected cognitively and demonstrated the most robust 5hmC and metabolite changes, we investigated whether these alterations were responsive to beneficial changes in diet as previously shown in WT mice^22^. Many of the genomic regions and pathways that were significantly hypermethylated in E4 HFD mice were hypomethylated following the HFD → LFD intervention, suggesting that these genes may play a role in the functional impairments, and subsequent recovery. Similarly, a number of metabolites and pathways altered by HFD were also revealed during an untargeted metabolomics screen of HFD → LFD mice. Importantly, the three metabolic pathways most impacted by the intervention were purine metabolism, glutamate metabolism, and the PPP. These results point to a functional role for the aforementioned pathways, as they are significantly altered by both the chronic HFD and the dietary intervention.

Our results have several important implications: 1) the metabolic and cognitive dysfunction brought on by a HFD is magnified by E4, 2) is characterized by a distinct hippocampal epigenome and metabolome, 3) is responsive to a dietary intervention, and 4) may be driven by alterations in metabolic pathways of purine metabolism, glutamate, and the PPP. Together, these results suggest a susceptibility of E4+ carriers to metabolic and cognitive impairments brought on by a HFD, and may help guide development of novel therapeutic targets for AD and related dementias.

## Methods

### Experimental Animals, Biochemical Measures, and Cognitive Analyses

Female homozygous human E3 and E4 targeted replacement mice^50^ were fed a HFD (60% kcal from fat, Research Diets D12492) or ingredient-matched LFD (10% kcal from fat, D12450B) for 6 months, beginning at 9 months of age (tested at 15 months of age). T1D was induced by five days of sequential intraperitoneal injections of low-dose streptozotocin (STZ), beginning at 12 months of age (0.05 mg/g body wt in 0.05 mol/L citrate buffer, pH 4.5). T1D mice were tested 3 months after injection of STZ (tested at 15 months of age). Fasting plasma insulin (Millipore) was measured after a four hour fast. Glucose tolerance and glucose uptake were performed as described^22^. Object recognition task, spatial learning and memory in the water maze, and cued fear conditioning were assessed as described^22^. Procedures complied with the NIH Guide for the Care and Use of Laboratory Animals and with IACUC approval at OHSU.

### Untargeted Metabolomics

Hippocampi were dissected and homogenized in RIPA (500 μl). Metabolites were extracted from 100 μl of hippocampal homogenate and untargeted metabolomics was completed as described^22^,^51^. Metabolomics data was processed using MarkerView and Peakview software (AB SCIEX) and Metaboanalyst^52^. Metabolite identification was based on mass error (<30 ppm) and MS/MS fragment ions. Many metabolites were further confirmed using retention time and comparison to authentic standards ( ± 1 min) from an in-house library (IROA Technologies). LipidMaps, METLIN and HMDB databases were used for MS and MS/MS matching. Metaboanalyst heatmap and pathway analyses were performed as described^22^. For integrated pathway analyses, the parameters were set to ‘global test’ and ‘relative betweenness centrality’.

### DNA Hydroxymethylation Sequencing and Bioinformatics

DNA was isolated from 200 μl hippocampal homogenates (described above) and specific antibodies against 5-hydroxymethylcytosine (5hmC) were used to immunoprecipitate DNA preparations for hydroxymethyl-DNA immunoprecipitation (hMeDIP) as previously described^20^. Equal concentrations of individual hippocampal DNA samples were pooled to form 10 pools for hMeDIP analysis (2 pools/experimental group, consisting of 4–6 individual DNA samples/pool). Antibodies were used to precipitate genomic regions that are enriched for 5hmC, and following immunoprecipitation, high throughput genomic sequencing (Seq) was used to identify these enriched genomic regions. Immunoprecipitation, DNA isolation/preparation, and DIP-Seq library preparation were performed as described^20^. Libraries were sequenced on the NextSeq 2500 platform (OHSU Massively Parallel Sequencing Shared Resource). For hMeDIP-Seq analyses, 50 bp single read sequence data were mapped to the mouse reference genome (UCSC mm9) using the Bowtie algorithm using standard flags and allowing two mismatches. Sequences that map to a single location were selected and domains enriched for 5hmC were selected using a parameter-optimized Monte-Carlo-based segmentation algorithm, as previously described^20^. A 1,000 base pair sliding-window was selected based on iterative analyses that maximized the number of enriched regions. For statistical comparisons of hMeDIP biological samples, regions of methylation enrichment were merged and differences in methylation interrogated with FDR-adjusted chi-square statistics, as previously described^20^. hMeDIP DIP analyses were validated by replicating hmC DIP in a subset of samples and 5hmC enrichment was assessed using real-time PCR primers that target the DIP-Seq centroid of 5 gene-associated regions selected in an unbiased manner (Figure S6). Distribution of hMeDIP within various genomic regions and repetitive elements was analyzed using Fisher’s exact test. For statistical comparisons of hMeDIP biological samples, regions of methylation enrichment were merged and differences interrogated with FDR-adjusted chi-square statistics. Distribution of 5hmC within various genomic regions was analyzed using Fisher’s exact test. GO analyses involved the Database for Annotation, Visualization and Integrated Discovery (DAVID) v6.7. Functionally related terms that shared >75% of genes were considered redundant and removed.

### Statistical Analyses

All data are expressed as mean ± standard error. Multiple groups and/or multiple time points were analyzed using ANOVAs (Graph Pad Prism), or repeated measures ANOVA (time × groups) (SPSS). Statistical significance was determined using an error probability level of p < 0.05 corrected by a false discovery rate (FDR) analysis (Benjamini Hochberg method).

## Additional Information

**How to cite this article:** Johnson, L. A. *et al*. Apolipoprotein E4 and Insulin Resistance Interact to Impair Cognition and Alter the Epigenome and Metabolome. *Sci. Rep.* **7**, 43701; doi: 10.1038/srep43701 (2017).

**Publisher's note:** Springer Nature remains neutral with regard to jurisdictional claims in published maps and institutional affiliations.

## Supplementary Material

Supplementary Information

Supplementary DataSet

Supplementary Table1

Supplementary Table2
