# Supplementary material for: Apolipoprotein E4 and Insulin Resistance Interact to Impair Cognition and Alter the Epigenome and Metabolome
Source: Sci Rep. 2017 Mar 8;7:43701. doi: 10.1038/srep43701 (PMC5341123; doi:10.1038/srep43701)
Supplement: Supplementary Information [file srep43701-s1.pdf]

Apolipoprotein E4 and Insulin Resistance Interact to Impair Cognition and Alter the Epigenome and Metabolome

Lance A. Johnson, Eileen R.S. Torres, Soren Impey, Jan F. Stevens and Jacob Raber

Supplemental Figure Legends

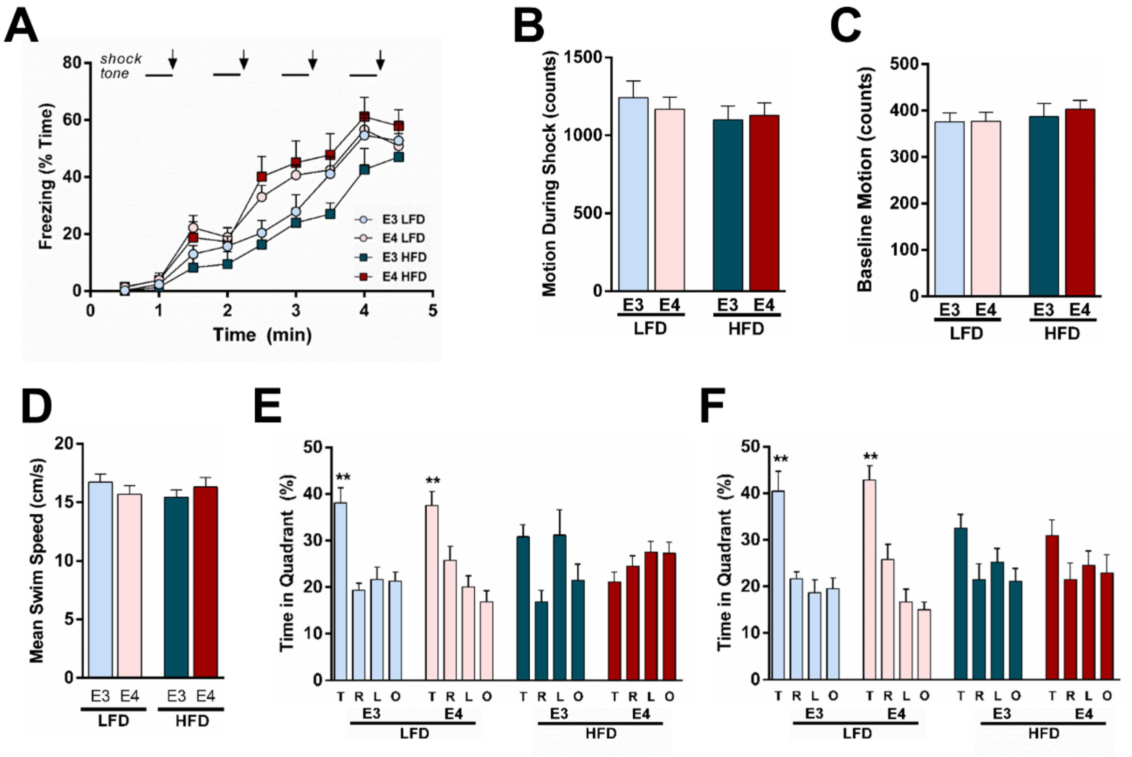

Supplemental Figure 1. Behavioral measures.

A) E3 mice acquire cued fear learning at a slower rate, but freezing is similar by the final minute of training. Mice were conditioned to associate a cue (tone) with a mild foot shock. (*n*=10-14) (*p* = 0.010 *APOE* effect, *p* = 0.630, diet effect, repeated measures ANOVA) (final minute, *p* = 0.608, ANOVA).

B) Responsiveness to the shock was similar among all groups. Responsiveness was assessed by measuring motion during the 2 second foot shock. (*n*=10-14) (*p* = 0.796, ANOVA)

C) Generalized fear does not differ between groups. Motion during the first minute (tone-free) of exposure to the novel environment of the cued fear chamber was measured. (*n*=10-14) (*p* = 0.724, ANOVA)

D) Swim speed during the water maze is similar among all groups. (*n*=10-14) (*p* = 0.643, ANOVA)

E-F) HFD impairs spatial memory. The accuracy of long-term spatial memory was measured by the percent time spent searching in the target quadrant during the first (G) and second (H) 24 hour memory probes. (*n*=10-14)

Note: For *E-F*, Target quadrant compared to all other quadrants (ANOVA followed by Tukey's multiple comparison test). T, target; R, right; L, left; O, opposite. Error bars represent mean ± SEM.

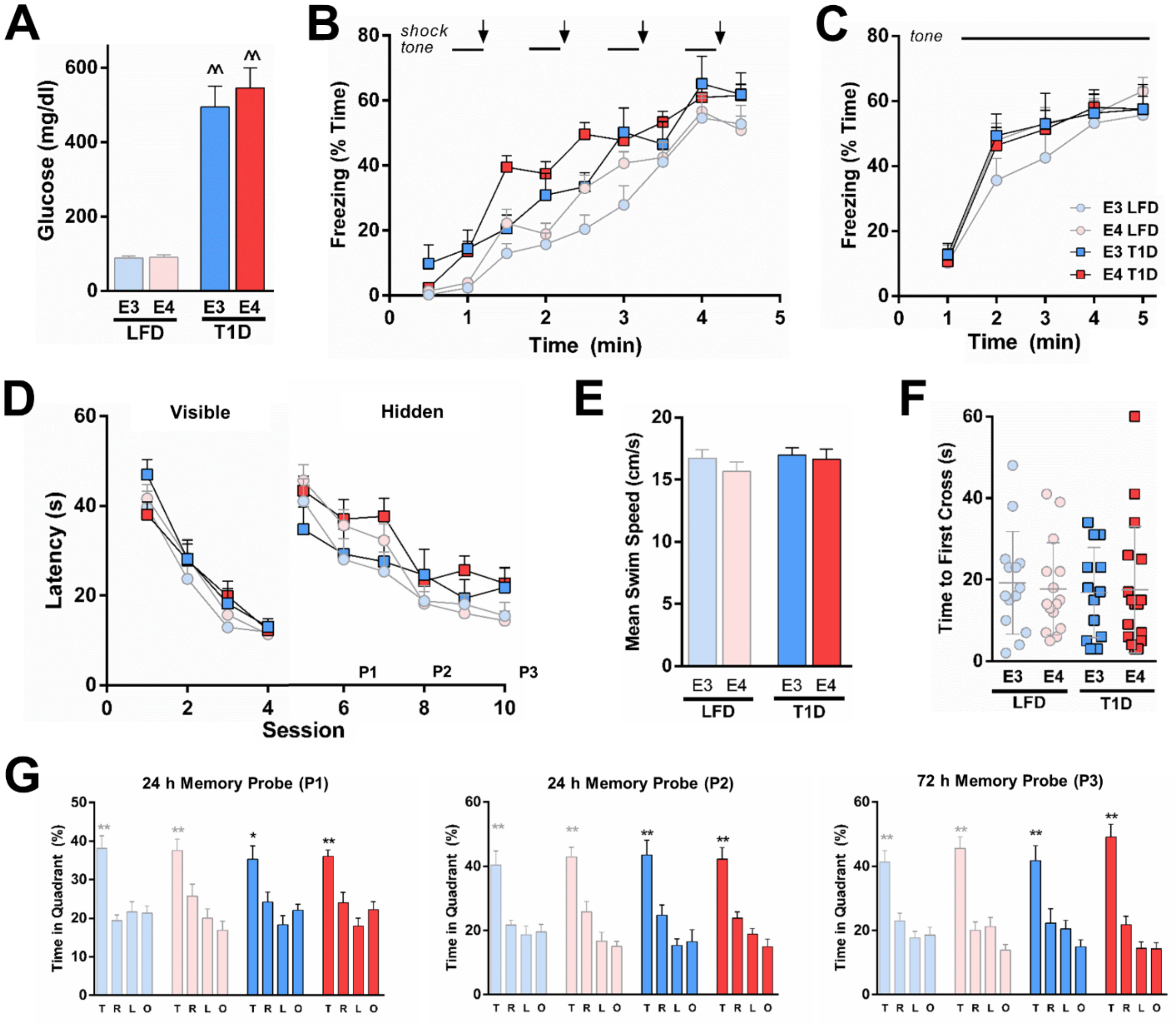

Supplemental Figure 2. Cognitive function is unaffected in a model of Type 1 Diabetes.

A) Severe hyperglycemia in E3 and E4 mice treated with STZ, a model of T1D. Blood glucose was measured following an overnight fast. **(n=7-10)**

B-C) Cued fear memory is unaffected in a model of T1D. Mice were conditioned to associate a cue (tone) with a mild foot shock (B). Cued memory recall (C) was assessed by measuring freezing behavior in response to the tone 24 h after training. **(n=13-17)**

D-E) Spatial learning and memory is unaffected in a model of T1D. Latency to find a visible (left) or hidden (right) escape platform (D) and swim speed (E) during the water maze. The timing of tests of long-term memory retention (shown in F-H) are noted (P1-P3). **(n=13-17)**

F-G) Long-term spatial memory is unaffected in a model of T1D. The accuracy of long-term spatial memory was measured by calculating the average time at which the mice first cross the target location (F) and the percent time spent searching in the correct (T, target) quadrant (G). **(n=13-17)**

Note: For *A*, ^^$p < 0.01$, compared to LFD (ANOVA followed by Tukey's multiple comparisons test); For *G*, *$p < 0.05$, **$p < 0.01$, Target quadrant compared to all other quadrants (ANOVA followed by Tukey's multiple comparisons test). E3 LFD and E4 LFD data (greyed out) is reproduced from Figures 1-2. Error bars represent mean ± SEM.

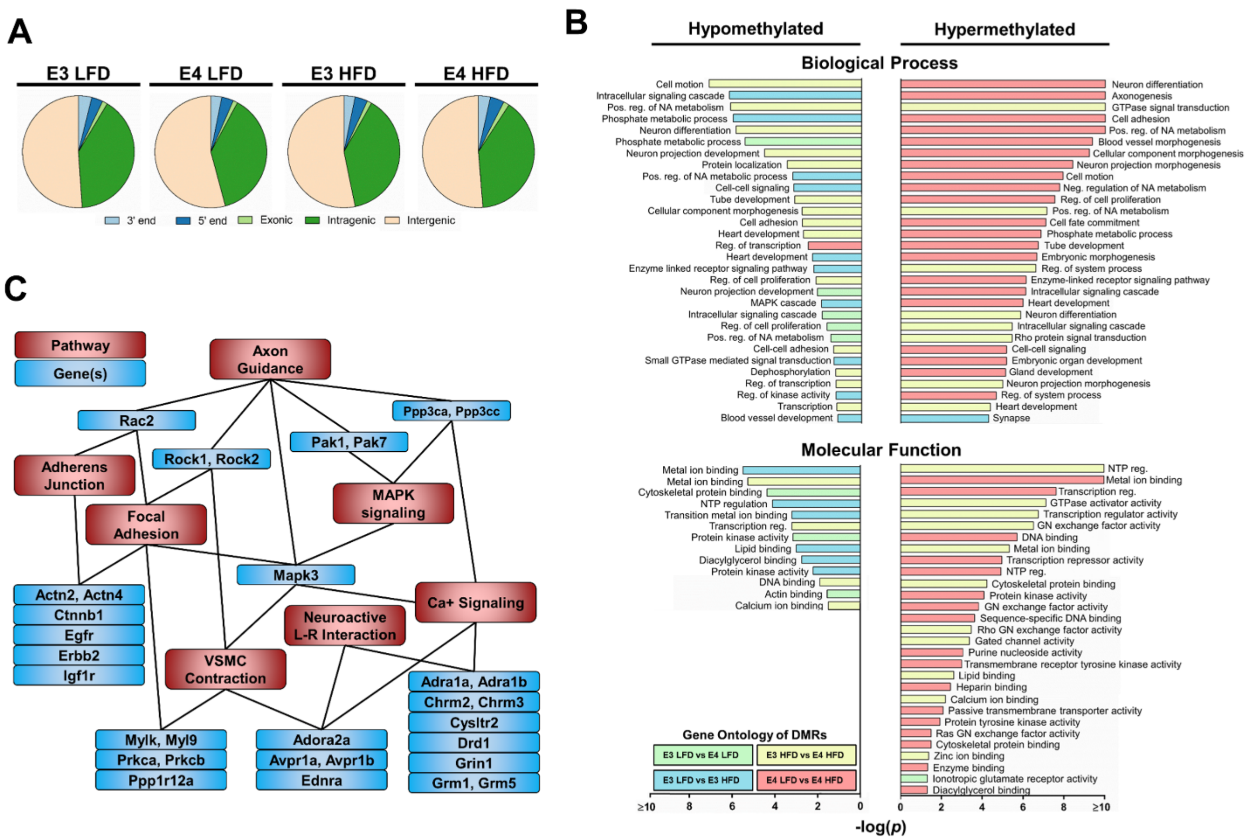

Supplemental Figure 3. 5hmC genomic annotation and gene ontology categorization.

A) 5hmC bases are located in similar genomic regions. Pie charts depict accumulation of 5hmC DIP-Seq signal in the indicated genomic regions (DIP-Seq counts per million repeat sequences). Data that matched multiple categories were matched exclusively to the closest annotation. (Distribution within various genomic regions was analyzed using Fisher's exact test, $p > 0.630$).

B) Gene ontology reveals 5hmC enrichment for categories related to neural function, development and metabolism. Bar graph depicts the top 30 most significantly enriched gene ontology terms in the indicated comparisons of DMRs based on biological process and molecular function categories (FDR-adjusted –log[$p < 0.01$]).

C) Genes that co-regulate multiple biological pathways were uniquely hydroxymethylated in E4 HFD mice. Diagrams depict selected that were significantly associated with altered 5hmC levels in E4 HFD mice.

Abbreviations: DNA, Deoxyribonucleic acid; GN, guanyl-nucleotide; L-R, ligand-receptor; NA, nucleic acid; Neg., Negative; Pos., positive; Reg., Regulation; NTP, Nucleoside-triphosphate

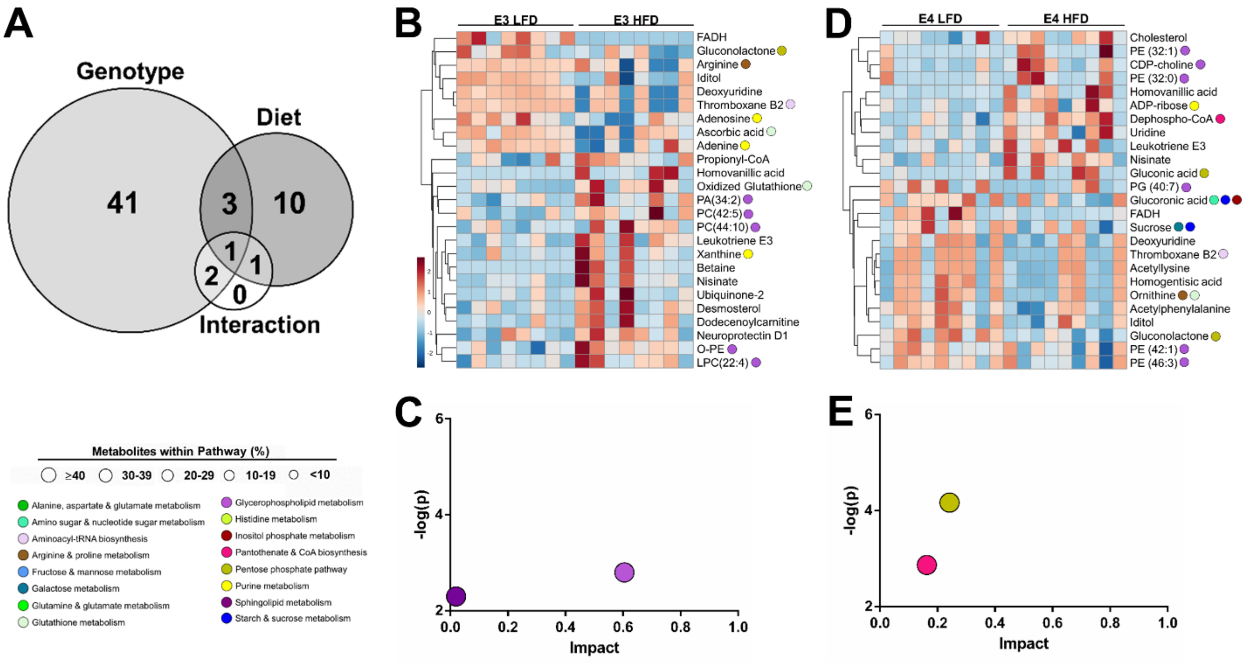

Supplemental Figure 4. HFD alters the hippocampal metabolome differently in E3 and E4 mice.

A) The majority of significantly altered metabolites differ by *APOE* genotype. Venn diagram depicts overlap between metabolites affected by *APOE* genotype, HFD, and/or an interaction between the two ($p < 0.05$, ANOVA).

B-E) Different metabolic pathways are altered by HFD in E3 and E4 mice. Hierarchical clustering of the metabolites most significantly altered by HFD in E3 LFD (B) and E4 (D) mice. Color in the heat map reflects the relative metabolite abundance level, with red being higher, and blue lower, than the mean value. Colored circles denote the metabolic pathway(s) in which each metabolite plays a role. A global view of the metabolome was created using a pathway impact analysis (C,E), which reflects key nodes in pathways that have been significantly altered by HFD in E3 (C) or E4 (E) mice. Circle size reflects the percentage of all metabolites within a given pathway that are represented. (n=8-9).

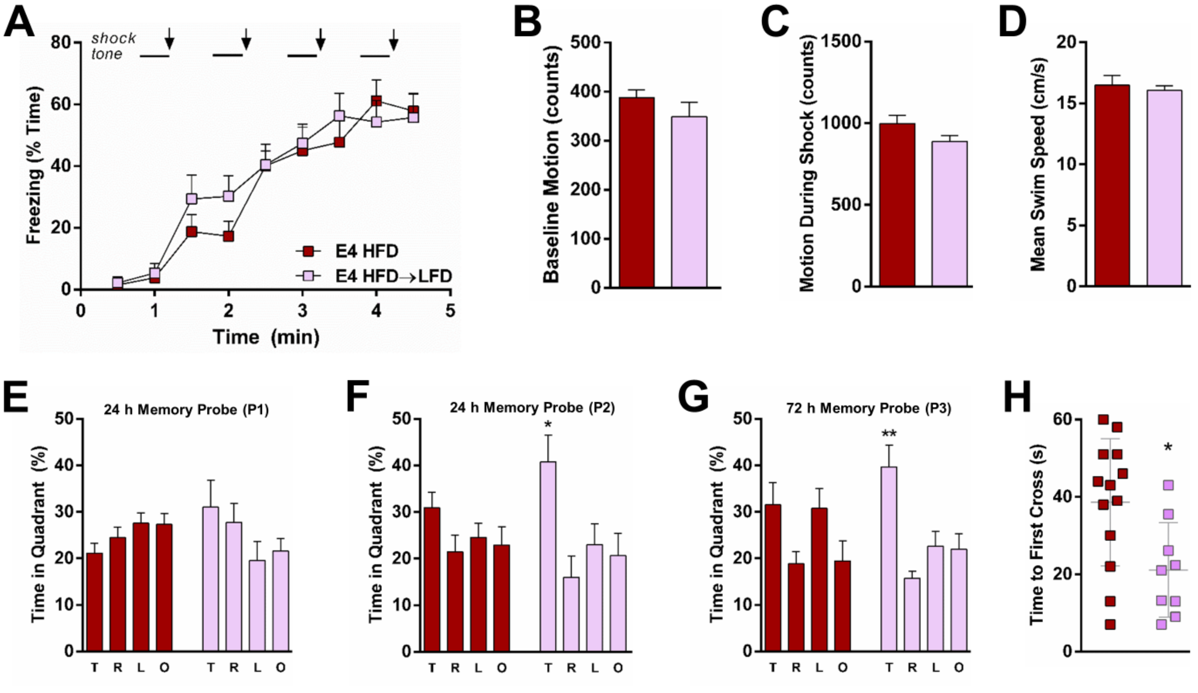

Supplemental Figure 5. Diet intervention rescues spatial memory in E4 HFD→LFD mice but does not affect acquisition of cued fear or swim speed.

A) Fear learning is unaffected by the diet intervention. Mice were conditioned to associate a cue (tone) with a mild foot shock. (*n*=9-13).

B-C) E4 HFD and E4 HFD→LFD mice show a similar baseline motion (prior to the tone on the training day) and responsiveness to the shock. (B). Responsiveness to the shock is measured by measuring motion during the 2-second foot shock (C). (*n*=9-13).

D) Swim speed during the water maze is unaffected by the diet intervention. (*n*=9-13).

E-H) Spatial memory is rescued in E4 HFD→LFD mice. Time spent searching in each quadrant of the water maze during the 24 hr (E,F) and 72 hour (G), and average time at which the mice first cross the target location during the memory probes (H). (*n*=9-13).

Note: For *E-G*, *$p < 0.05$, **$p < 0.01$, Target quadrant compared to all other quadrants (ANOVA followed by Tukey's multiple comparison test); For *H,* *$p < 0.05$ compared to E4 HFD (two-tailed Student's t test). Error bars represent mean ± SEM. E4 HFD data is reproduced from Figure 2.

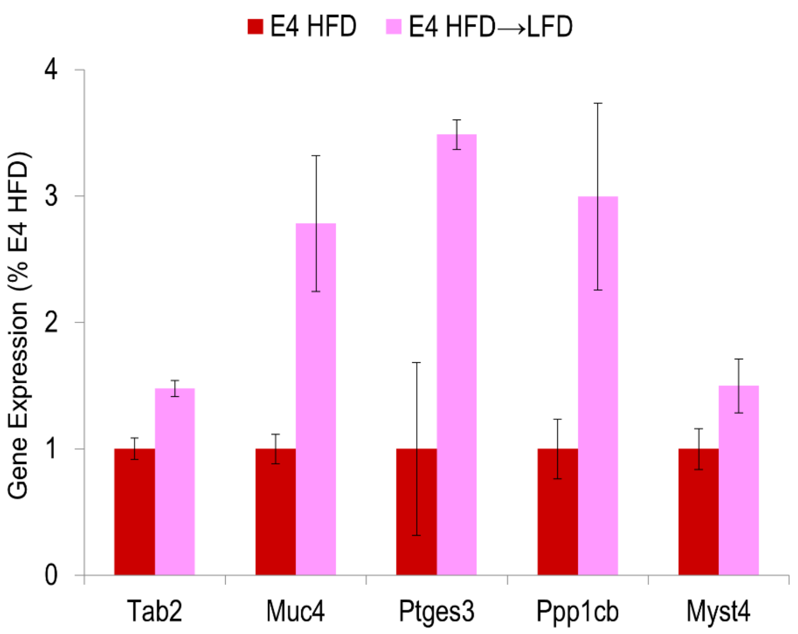

Supplemental Figure 6. 5hmC DIP validation of E4 vs E4 HFD→LFD significantly up-regulated DIP-Seq regions.

Pooled DNA for each condition was subjected to replicate 5hmC DIP (n=4). 5hmC enrichment was assessed using real-time PCR primers that target the DIP-Seq centroid of 5 gene-associated regions selected in an unbiased manner (ranked significance). * $p < 0.05$, ** $p < 0.01$.
